# Intragastric Carbon Dioxide Release Prolongs the Gastric Residence Time of Postprandially Administered Caffeine

**DOI:** 10.3390/pharmaceutics15031012

**Published:** 2023-03-22

**Authors:** Stefan Senekowitsch, Constantin Foja, Toni Wildgrube, Philipp Schick, Christoph Rosenbaum, Julius Krause, Friederike Brokmann, Marie-Luise Kromrey, Stefan Engeli, Werner Weitschies, Michael Grimm

**Affiliations:** 1 Department of Biopharmaceutics and Pharmaceutical Technology, Center of Drug Absorption and Transport (C_DAT), University of Greifswald, Felix-Hausdorff-Str. 3, 17487 Greifswald, Germany; 2 Department of Diagnostic Radiology and Neuroradiology, University Hospital Greifswald, Ferdinand-Sauerbruch-Straße, 17475 Greifswald, Germany; 3 Institute of Pharmacology, Center of Drug Absorption and Transport (C_DAT), University Medicine Greifswald, Felix-Hausdorff-Str. 3, 17487 Greifswald, Germany

**Keywords:** salivary tracer technique, caffeine, effervescent granules, in vivo study, carbon dioxide, sparkling water

## Abstract

Sparkling water is said to increase gastric motility by the release of carbon dioxide, thereby potentially affecting the pharmacokinetics of orally administered drugs. The hypothesis of the present work was that the induction of gastric motility by intragastric release of carbon dioxide from effervescent granules could promote the mixing of drugs into the chyme under postprandial conditions, resulting in a prolonged drug absorption. For this purpose, an effervescent and a non-effervescent granule formulation of caffeine as a marker for gastric emptying were developed. In a three-way crossover study with twelve healthy volunteers, the salivary caffeine pharmacokinetics, after administration of the effervescent granules with still water and the administration of the non-effervescent granules with still and sparkling water, were investigated after intake of a standard meal. While the administration of the effervescent granules with 240 mL of still water led to a significantly prolonged gastric residence of the substance compared to the administration of the non-effervescent granules with 240 mL still water, the application of the non-effervescent granules with 240 mL sparkling water did not prolong gastric residence via mixing into caloric chyme. Overall, the mixing of caffeine into the chyme following the administration of the effervescent granules did not seem to be a motility mediated process.

## 1. Introduction

Usually, oral dosage forms shall be ingested together with water. The term “water” can be ambiguous. There are different kinds of water regarding its origin, mineral content, or carbonic acid content, among other things. In Germany, water with a high carbonic acid content makes up 41.1% of sold bottled mineral water [1]. While there are no specific studies about the ingestion habits of oral medicines of patients regarding the carbonation of the co-administered water, it is likely that a certain percentage of patients take their medicines together with sparkling water instead of still water. The high amount of carbonic acid in sparkling water leads to a release of carbon dioxide in the gastrointestinal tract, which could affect the gastrointestinal physiology. 

Van den Abeele et al. showed that the co-administration of sparkling water in the fasted state can lead to a faster and less variable absorption of paracetamol, most likely due to a faster in vivo tablet disintegration and the induction of gastric motility [2]. An increase in gastric motility after the intake of soda was also observed using bowel sound-based methods in several studies [3,4,5]. Additionally, the distribution of solids and liquids between the proximal and distal parts of the stomach under fed conditions is different after the application of sparkling water compared to still water [6]. 

Due to the different physiological conditions of the stomach under fed conditions, the gastric emptying of drugs and thus the onset of drug absorption can differ from intake under fasted state conditions. Immediate release formulations taken with water in the fed state can lead to different scenarios of onset and a course of drug uptake [7]. The emptying process of non-caloric liquids can usually be described by first-order kinetics. It was shown that 240 mL of still water are usually emptied from the stomach within 15–45 min under fasted and fed state conditions [8,9,10,11]. Typically, in the fed state, non-caloric fluids are not mixed with the chyme, but follow a “shortcut”, the so-called stomach road (“*Magenstrasse*”). Drug content that is not mixed with the chyme follows the direction of the stomach road and thus a fast onset of plasma concentrations can be observed. In contrast, drug content that is mixed with the chyme can be expected to be emptied from the stomach at a slower rate, as the content of the fed stomach is emptied by a constant, calorie-dependent rate of approximately 2–4 kcal/min [8].

An increase of the gastric motility by carbon dioxide might induce a pronounced mixing effect. This might lead to a reduction of the effect of the stomach road and thereby cause a delay of the emptying of the administered drug. Thus, a dosage form that releases carbon dioxide in the stomach could potentially be used to enhance the gastric residence time of drugs under fed intake conditions.

It was the purpose of this study to investigate the influence of the intragastric release of carbon dioxide on the gastric residence time of the model drug caffeine under fed conditions. For this purpose, an effervescent and a non-effervescent granule formulation were developed. Both formulations contained caffeine, which is used as an established salivary marker to non-invasively evaluate gastric emptying [12]. Oral contamination with caffeine should be avoided by the administration of the granules in a thin water-soluble polymer bag. We planned to investigate salivary caffeine pharmacokinetics in healthy volunteers after administration of the effervescent granules together with still water and the administration of the non-effervescent granules with still as well as with sparkling water. 

## 2. Materials and Methods

### 2.1. Materials

Caffeine, citric acid (anhydrous), anhydrous glycerol, black iron oxide (E172), lactose monohydrate, and blue food coloring (E131) were obtained from Caesar & Loretz GmbH (Hilden, Germany). Macrogol 4000, magnesium stearate and Aerosil^®^ 200 have been purchased from Fagron GmbH & Co. KG (Barsbüttel, Germany). Mannitol (Mannogem^®^ EZ) as well as sodium hydrogen carbonate (EfferSoda^®^ 12) were obtained from Lehmann&Voss&Co. (Hamburg, Germany). Microcrystalline Cellulose (VIVAPUR^®^ 101) was purchased from JRS PHARMA GmbH & Co. KG (Rosenberg, Germany). Polyvinylpyrrolidone (PVP) (Kollidon^®^ 90F) and Polyvinyl alcohol-polyethylene glycol copolymer (Kollicoat^®^ IR) were ordered from BASF SE (Ludwigshafen, Germany). Polyvinyl alcohol (PVA) 4-88 Parteck^®^ MXP was supplied by Merck Darmstadt GmbH (Darmstadt, Germany). 

The components of the meal consisting of bacon (Tulip bacon, Tulip Food Company, Hamburg, Germany), butter (Meggle Alpenbutter, MEGGLE GmbH & Co. KG, Wasserburg, Germany), white toast (Sammy’s Super Sandwich, Harry-Brot GmbH, Schenefeld, Germany), eggs (Freilandeier Größe L, Poseritzer EierHOF, Poseritz, Germany), hash browns (Gut&Günstig Rösti-Ecken, Gut&Günstig brand of the EDEKA group, Hamburg, Germany), whole milk (Gut&Günstig H-Vollmilch 3.5% Fett, Gut&Günstig brand of the EDEKA group, Hamburg, Germany), as well as still (Gerolsteiner Naturell, Gerolsteiner Brunnen GmbH & Co. KG, Gerolstein, Germany) and sparkling water in glass bottles (Gerolsteiner Sprudel, Gerolsteiner Brunnen GmbH & Co. KG, Gerolstein, Germany) were purchased at a local supermarket.

Hydrochloric acid for the preparation of dissolution media was purchased from Walter-CMP GmbH & Co. KG (Kiel, Germany). Sodium hydroxide has been ordered from Honeywell Fluka (Seelze, Germany). Sodium chloride was obtained from Sigma-Aldrich (Steinheim, Germany). Potassium dihydrogen phosphate was purchased from neoLab Migge GmbH (Heidelberg, Germany). 

All solvents used for LC-MS analysis, i.e., water, methanol, formic acid, and acetonitrile were purchased in LC-MS grade from VWR international (Fontenay-sous-Bois, France).

### 2.2. Methods

#### 2.2.1. Formulation of the Effervescent and the Non-Effervescent Granules

As can be seen in Table 1, the composition of both formulations was the same, except for a higher percentage of microcrystalline cellulose (MCC) and lactose monohydrate to compensate for the absence of citric acid and sodium hydrogen carbonate. The manufacturing process of both formulations was the same. The pulverized ingredients were standardized by sieving through a sieve with a mesh size of 355 up to 500 µm. Citric acid, sodium hydrogen carbonate, and MCC were dried for 50 min at 50 °C in a drying cabinet (FDL 115–230 V, BINDER GmbH, Tuttlingen, Germany). Afterwards, the dried powder was extruded using a twin-screw extruder (Three-Tec ZE12, Three-Tec GmbH, Seon, Switzerland) with 12 mm diameter screws with an L/D of approximately 20:1, four heated zones, and a water-cooled feeding zone. The extrusion was performed without a nozzle plate at a rotation speed of 100 rpm. The powder mixture was fed using a Flat-Tray Feeder (ZD 9 FB, Three-Tec GmbH, Seon, Switzerland). 

The filling rate of the extruder by the Flat-Tray Feeder was set to 15% and the filling segment was cooled to 20 °C. All four heated zones were tempered to 70 °C. The hot granules were collected on glass containers. After a cooling period, the granules were pressed through a sieve with a mesh size of 1000 µm and 500 µm successively and collected by a sieve with a mesh size of 355 µm.

The distribution of the size of the granule particles was determined by the use of a sieve tower (Retsch AS 200 basic, Retsch GmbH, Haan, Germany). The granule particles were assigned in three fractions with sizes of 1.7–2.0 mm, 0.5–1.7 mm, and 0.0–0.5 mm, respectively. The second fraction was used for further manufacturing steps.

#### 2.2.2. Preparation and Filling of a Granule Carrier

Due to the application of the salivary tracer technique, oral caffeine contamination during the intake of the granules had to be avoided. Hard capsules were precluded due to limited filling capacity and too long and variable disintegration times in vivo when taken under fed conditions [13,14]. To solve this problem, a small bag made out of a thin film was manufactured. The film consisted of a mixture of 10% PVA (4-88 Parteck^®^ MXP), 20% Kollicoat^®^ IR, 5% anhydrous glycerol, 64% deionized water, and 1% blue food colorant (E131).

In order to produce the film, the blue dye, anhydrous glycerol, and deionized water were mixed at a speed of 500 rpm by a magnetic stirrer (IKA^®^ RCT basic, IKA-Werke GmbH & CO.KG, Staufen, Germany) in a threaded bottle. PVA and Kollicoat^®^ IR were added successively. The threaded bottle was closed and the mixture was stirred at 80 °C at a speed of 200 rpm for five hours. The obtained solution was transferred to falcon tubes (Sarstedt, Nümbrecht, Germany) and centrifuged (Eppendorf Centrifuge 5702R, Eppendorf SE, Hamburg, Germany) at a speed of 4400 rpm (3000× *g*) for 60 min in order to remove air bubbles.

Subsequently, a test tube (external diameter 16.5 mm) was dip-coated into the polymer solution and taken out again for drying. This procedure was repeated twice, to create a stable film with a reproduceable form. The film could be stripped off the test tube and was then filled with 1.5 g of the granules (corresponding to 100 mg caffeine) and sealed with a small amount of polymer solution.

#### 2.2.3. In Vitro Testing of the Effervescent Formulations

The dissolution behavior of the prepared formulations was investigated in a compendial paddle apparatus (PT-DT70, PharmaTest Apparatebau AG, Hainburg, Germany). All experiments were performed in 900 mL medium at 37 °C and a rotational speed of 75 rpm. As test media, Simulated Gastric Fluid sine pepsin (SGF sp) with a pH of 1.2 and 50 mM phosphate buffer pH 6.8 were used. These media were prepared according to the requirements of Ph. Eur. 10.7. 

The quantification of the caffeine concentration was performed using a UV/VIS-spectrophotometer (Cary 60 UV-VIS, Agilent, Santa Clara, CA, USA) connected with fiber optics. The absorption of the solution at the wavelength of 272.0 nm and 500.0 nm (as baseline correction) was measured every minute. The gap width of the fiber optic tips was 1 mm.

Additionally, the dissolution behavior of the granules in the carrier was examined under more physiologically relevant parameters using the GastroDuo, a biorelevant dissolution test device [15]. 

The GastroDuo is designed as a flow-through system. It is possible to simulate various physiologically relevant parameters influencing gastric emptying, such as temperature changes, dynamic pH profiles, pressure events, and realistic movements. The central compartment is the gastric cell, in which the dosage form to be tested is placed at the beginning and several peristaltic pumps ensure the inflow and outflow to maintain a constant volume within the gastric cells. Additionally, pH-electrodes (InLab^®^ Expert Pro, METTLER TOLEDO, Greifensee, Switzerland) and fiber optic probes (Cary 50, Varian Inc., Mulgrave, Australia) are installed in the model to measure resulting drug concentrations and characterize the media. For a more detailed description of the model we refer to a previous publication [15].

**Figure 1:**
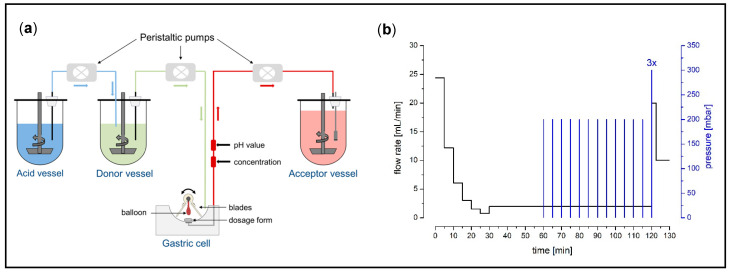
Schematic of the biorelevant dissolution model GastroDuo (reprinted with permission from Schick et al. [15]. Copyright 2019 American Chemical Society) (**a**) and the test program for fed state simulation (**b**). The black line represents the flow rate and the blue lines represent pressure events every 5 min. Furthermore, slow movements were carried out every 20 s, from 60 to 120 min.

The program used to assess the dissolution of the effervescent granule under fed conditions as well as the GastroDuo are shown in Figure 1. The gastric cell was perfused by mixture of 50 mM citrate and 50 mM phosphate buffer pH 4.5 that was contained in the donor vessel. By the addition of SGF sp pH 1.2, this buffer mixture was acidified over time. Within the initial 30 min, the rapid emptying of 240 mL of water (so-called stomach road) was simulated by flow rates, which decreased by a first-order kinetic every five minutes. Onwards, a low basal flow rate of 2 mL/min was applied to account for secretion processes in the stomach. From 60 to 120 min, the balloons were inflated with “low” pressures of 200 mbar every 5 min, followed by a slow movement of the blades, to simulate gastric peristalsis. After 120 min, the recurrence of the fasted state pattern was simulated by the execution of high pressures of 300 mbar and rapid movements of the blades in order to simulate the occurrence of migrating motor complex (MMC) phase 3 (so-called housekeeping waves).

#### 2.2.4. In Vivo Study

The in vivo study was performed in a crossover design with three study arms: twelve healthy young volunteers (4 women and 8 men) aged from 23 to 30 years were enrolled. The study was not designed for a balance of sexes. Inclusion and exclusion criteria were closely adapted to EMA and FDA guidance for bioequivalence studies [16,17]. Good health and exclusion of pregnancy in women were ascertained before inclusion into the study. Known problems in swallowing larger monolithic dosage forms led to exclusion of candidates because large granule carriers were administered in this study. All volunteers gave their written informed consent prior to the study. The study procedures were in accordance with the Declaration of Helsinki (2013, Fortaleza, Brazil) and the Professional Code for Physicians in Germany (amended 2015 in Frankfurt, Germany). The ethics committee of the University Medicine Greifswald approved the study protocol and all related documents (Registration number BB 073/22). The study has been registered at the German Clinical Trials Register under ID: DRKS00031508.

72 h before intake of the granules, volunteers had to abstain from caffeine-containing products. The consumption of food and beverages prior to and during the study day was restricted as well. On the study day, the volunteers were allowed to ingest a light breakfast that did not contain more than 500 kcal, not later than 6 h before the ingestion of the high-caloric, high fat standard meal at noon. Caloric drinks were allowed until 3 h and non-caloric drinks until 90 min prior to the study, respectively. The participants presented to the Clinical Research Unit of the Centre of Drug Absorption and Transport (C_DAT) in Greifswald 10 min prior to the start of the ingestion of the standard meal. 

In the three study arms, the participants had to ingest a high caloric, high fat meal within 15 min. The composition of the meal was in accordance with the recommendation for a high caloric meal of the FDA guidance for assessing the effect of food on drugs [17]. It consisted of two slices of bacon, two slices of white toast with butter, two fried eggs in butter, 113 g of hash brown potatoes, and a glass (240 mL) of whole milk. The total energy content of the meal amounted to 800–1000 kcal. 

The formulations were administered 30 min after starting to eat, either with 240 mL still water (study arm 1: effervescent granules; study arm 2: non-effervescent granules) or 240 mL sparkling water (study arm 3: non-effervescent granules). The glass bottles of the sparkling water were opened immediately before administration to the volunteers to ensure a consistent carbonic acid content. Five minutes prior to the ingestion of the standard meal and 5 min prior to the application of the dosage form, the participants had to provide a blank saliva sample. Over the following six hours the volunteers had to collect saliva samples at 2.5, 5, 10, 15, 20, 25, 30, 45, 60 min, and onwards after every 15 min. Ingestion of food and drinks was prohibited during this period. Four hours after the intake of the dosage form, the participants received 240 mL of still water. In addition, the participants were asked to refrain from strenuous physical exercise.

Three different sequences of the study arms (1 → 2 → 3; 2 → 3 → 1; 3 → 1 → 2) were defined prior to randomization. The volunteers were allocated to these sequences by block randomization. For this, 4 blocks with 3 volunteers each were created. In these blocks, the volunteers were ranked by the value of random numbers that were generated with Microsoft Excel 2019 (Microsoft Corporation, Redmont, WA, USA) using the function RAND(). Depending on the rank, the volunteers were allocated to the corresponding sequence of the study arms: rank 1: 1 → 2 → 3; rank 2: 2 → 3 → 1; rank 3: 3 → 1 → 2. 

#### 2.2.5. Determination of Salivary Caffeine

Caffeine is an established marker substance for gastric emptying of liquids [12]. It is highly soluble at gastrointestinal pH-values and highly permeable (BCS class I). Thus, the rate limiting step of the absorption of caffeine is the emptying of the drug from the stomach. As salivary caffeine kinetics correlate with plasma concentration profiles and there is a constant saliva–plasma–concentration ratio of caffeine, the absorption of caffeine can be investigated by determining its salivary concentrations [18,19]. Therefore, in this study, the salivary pharmacokinetics of caffeine could be applied indirectly to evaluate the gastric residence time of caffeine. A slow and continuous increase of salivary caffeine concentration would indicate a slow release of the drug from the stomach.

For the sampling of saliva, the volunteers had to spit into two milliliters SafeSeal microtubes (Sarstedt, Nümbrecht, Germany). Stimulating the saliva flow by chewing on parafilm, using citric acid or other commonly applied techniques was not allowed. The saliva samples were stored at −80 °C until further preparation and analysis. Analysis of the samples was performed with a LC-MS/MS method that was validated according to the Guideline on bioanalytical method validation of the EMA [20]. For this, the saliva samples were thawed at room temperature, vortexed, and centrifuged at 13 000 rpm for 10 min (Biofuge pico, Heraeus, Hanau, Germany). 100 µL of the supernatant was transferred into 1.5 mL microtubes (Sarstedt, Nümbrecht, Germany), spiked with 25 µL Internal Standard solution (d_9_-caffeine 4.0 µg/mL) and vortexed (IKA^®^ VORTEX 2, IKA-Werke GmbH & CO.KG, Staufen, Germany). Subsequently, the proteins of the saliva were precipitated by an addition of 200 µL acetonitrile (containing 1% formic acid). After vortexing, the samples were centrifuged at 13,000 rpm for 10 min again. The supernatant was transferred into 1.5 mL HPLC glass vials (VWR International GmbH, Darmstadt, Germany) and analyzed by a LC-MS 8060 system (Shimadzu Corporation, Kyoto, Japan).

#### 2.2.6. Statistics

To account for occasional contamination of the blank saliva sample with caffeine, due to volunteers not complying with the caffeine restrictions prior to the study days, the caffeine concentration of the blank saliva sample (t = −5 min) was subtracted from all following salivary caffeine concentrations for statistical analysis. In the case of oral contamination with caffeine, apparent by an initial peak at the first sampling point with subsequent decline of concentration, all following salivary caffeine concentrations were excluded from determining c_max_, and calculation of AUC and mean caffeine concentration profile until the next minimal concentration value was reached. The AUC was calculated by applying the linear trapezoidal rule using Microsoft Excel 2019.

The pharmacokinetic parameters c_max_, t_max,_ AUC_0–120_, and AUC_0–360_ were statistically compared for all three study arms. It was checked if the datasets were normally distributed. For this purpose, a Kolmogorov–Smirnov test and a D’Agostino and Pearson omnibus normality test were applied. If one dataset did not comply with normal distribution, all datasets were compared non-parametrically. If this was the case, a non-parametric Friedman test with a Dunn’s post-hoc test were used. For normal distributed datasets, a one-way ANOVA with a Tukey post-hoc test were applied. All statistical evaluations were performed with GraphPad Prism 5 (GraphPad Software, Inc., San Diego, CA, USA).

## 3. Results

### 3.1. Final Products

**Figure 2:**
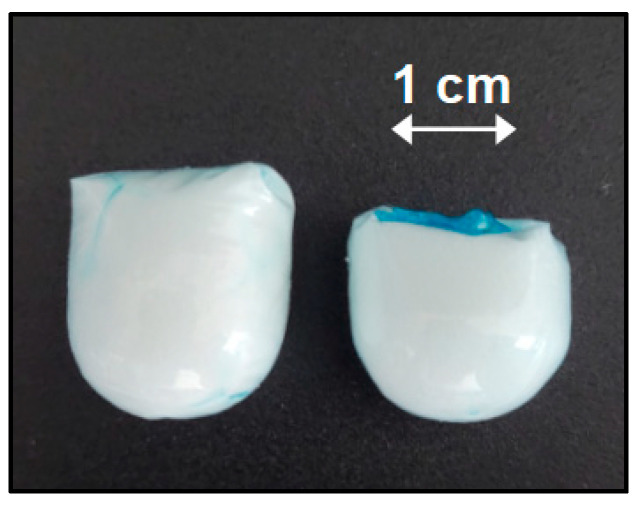
Filled granule carrier. **Left**: non-effervescent granules. **Right**: effervescent granules.

The final granules packaged in the carriers are shown in Figure 2. The carrier filled with the effervescent granule had a cylindrical shape with a length of about 17.5 mm and a diameter of approximately 15 mm., while the carrier that contained the non-effervescent granules had a size of 18.7 mm (length) × 16.5 mm (diameter). The larger proportions of the non-effervescent granules were mainly attributed to a lower bulk density due to the higher amount of MCC.

### 3.2. In Vitro Dissolution

**Figure 3:**
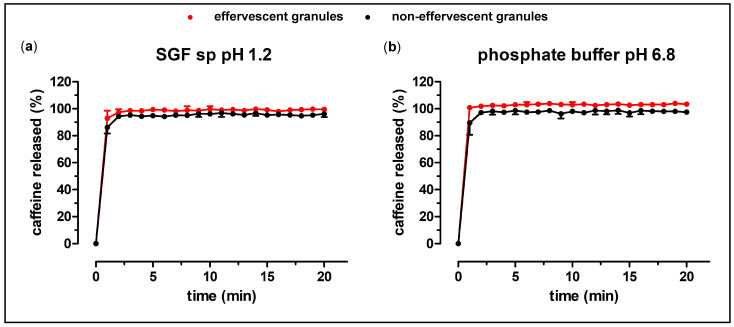
Dissolution profiles of the effervescent (red) and the non-effervescent granules (black) in 900 mL SGF sp pH 1.2 ((**a**), n = 6, mean ± standard deviation) and in 900 mL 50 mM phosphate buffer pH 6.8 at 37 °C ((**b**), n = 6, mean ± standard deviation) in the compendial paddle apparatus.

**Figure 4:**
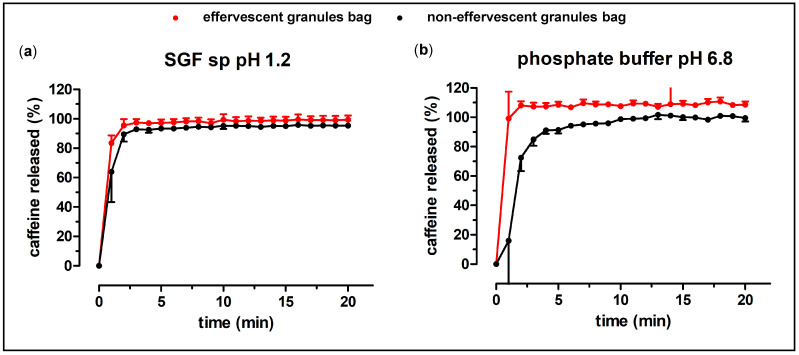
Dissolution profiles of the granule bags, filled with the effervescent (red) and the non-effervescent granules (black) in 900 mL SGF sp pH 1.2 ((**a**), n = 6, mean ± standard deviation) and in 900 mL 50 mM phosphate buffer pH 6.8 at 37 °C ((**b**), n = 6, mean ± standard deviation) in the compendial paddle apparatus.

For both formulations, compendial dissolution experiments were performed. Each bag contained 100 mg caffeine. Additionally, the granules have been characterized by dissolution experiments in the GastroDuo. The results of the compendial dissolution experiments of the granules are shown in Figure 3 and Figure 4.

The granules without the carrier showed a very rapid release in both of the tested media. The whole content of caffeine was released within two minutes, for both tested granules. As shown in Figure 4, the granule carrier did not delay the release of caffeine by a great extent. Notably, the dissolution of caffeine out of the packaged granule occasionally showed higher variability. The effervescent granule in the carrier in phosphate buffer showed a visibly higher finale caffeine concentration in comparison to the non-effervescent granules. 

**Figure 5:**
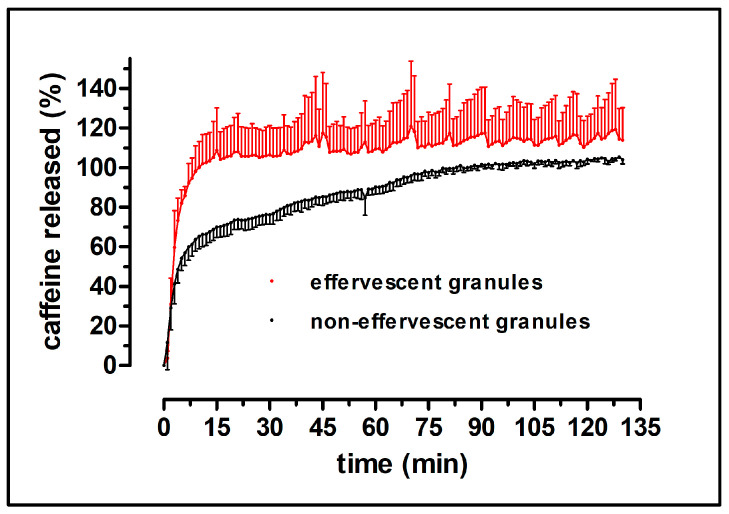
Dissolution profiles of the granule bags, filled with the effervescent (red) and the non-effervescent granules (black) in the GastroDuo at 37 °C (n = 3, mean ± standard deviation).

For the dissolution experiments in the GastroDuo, a mixture was applied of 50 mM citrate and 50 mM phosphate buffer pH 4.5 that was acidified by the addition of SGF sp pH 1.2 over time. Similar to the compendial dissolution test, a rapid release of caffeine from effervescent granules in the carrier could be observed. As can be seen in Figure 5, after 6 min, more than 85% of the caffeine dose was emptied into the acceptor vessel. The standard deviation was quite high in these experiments, probably due to the formation of CO_2_ bubbles interfering with the absorption measurements by the fiber optics. 

The non-effervescent granules in the carrier showed a slower release in the GastroDuo. After 15 min, 70% of the caffeine dose of 100 mg was emptied into the acceptor vessel. After 35 min, 80% release could be observed; while, after 83 min, 100% of the caffeine was found in the acceptor vessel.

### 3.3. In Vivo Study

**Figure 6:**
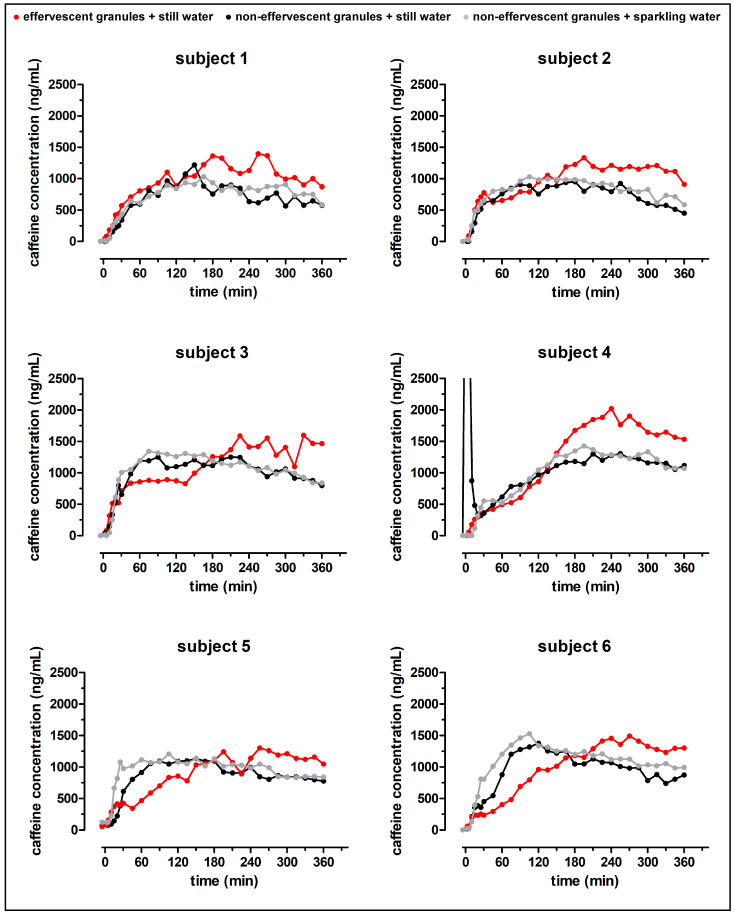

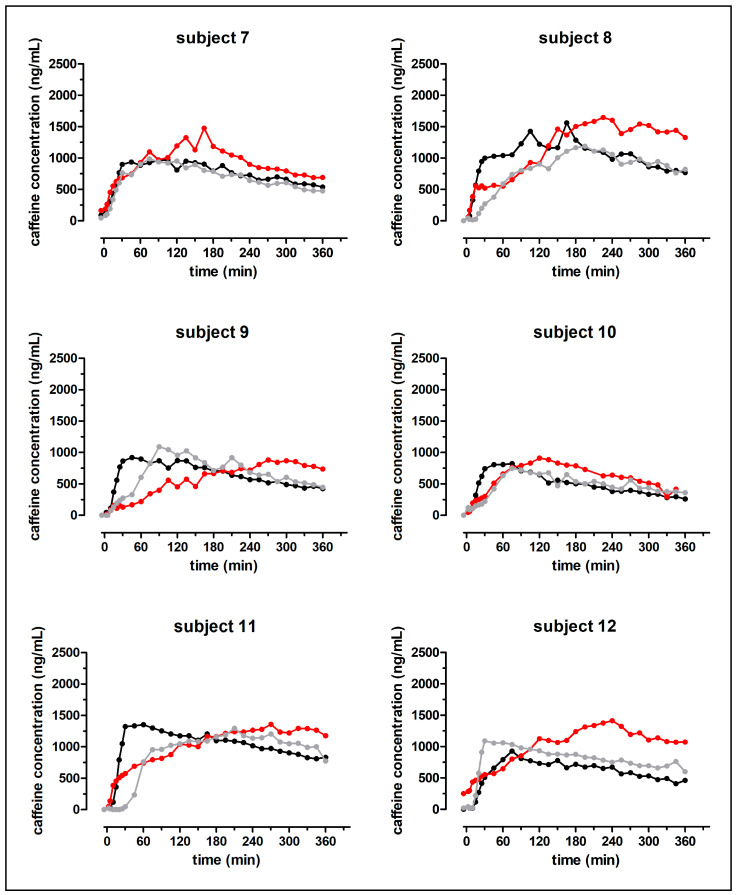
Individual salivary caffeine concentration profiles after the intake of the effervescent granules bag with still water (red), the non-effervescent granules bag with still water (black) and sparkling water (grey).

In total, 12 volunteers took part in the study. The demographic data of the volunteers are presented in Table 2. The individual salivary concentration profiles are given in Figure 6. As can be seen, noteworthy oral contamination, due to the disintegration of the polymer film in the oral cavity, was only observed for subject 4 after the application of the non-effervescent granules with 240 mL still water. Obviously, not all subjects did comply with the caffeine fasting regulations. The blank saliva (t = −5) of subjects 5 and 7 showed significant caffeine concentrations in all study arms, while for subject 12, this was only true for the application of the effervescent granules with still water. As described earlier, for statistical analysis, these blank caffeine concentrations were subtracted from the following values.

**Figure 7:**
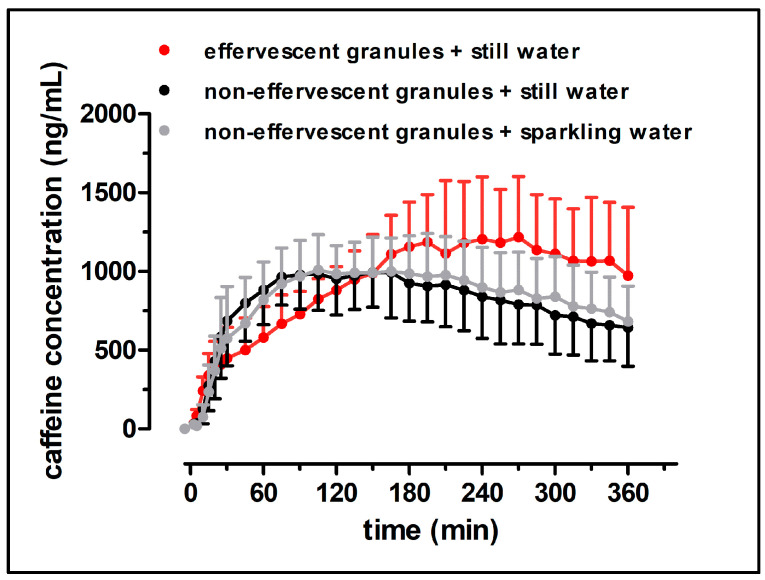
Mean salivary caffeine concentration profiles after the intake of the effervescent granules bag with still water (red), the non-effervescent granules bag with still water (black) and sparkling water (grey) (n = 12, mean ± standard deviation).

The mean salivary caffeine profiles after intake of the formulations are shown in Figure 7. Overall, the mean salivary caffeine concentration profiles were very similar after the application of the non-effervescent granules, regardless of the carbon dioxide content of the co-administered water. Noticeably, the mean profile after the application of the effervescent granules was different from those after the application of the non-effervescent granules. After a fast initial rise of the salivary caffeine concentrations, a slower increase was observed.

**Figure 8:**
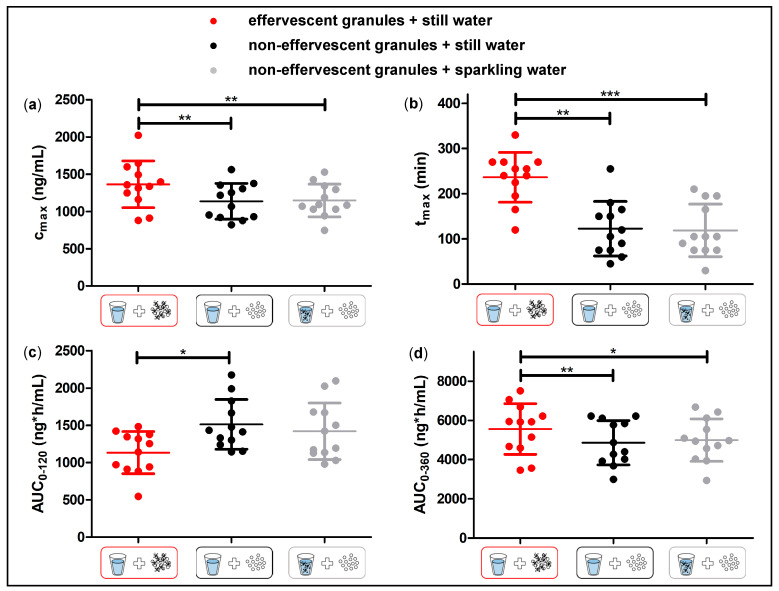
Salivary c_max_ (**a**), t_max,_ (**b**), AUC_0–120_ (**c**), and AUC_0–360_ (**d**) of caffeine after the application of the effervescent granules with still water (red) and the non-effervescent with still water (black) or sparkling water (grey) (n = 12, mean ± standard deviation, points represent individual values). Significant difference of c_max_, AUC_0–120_ and AUC_0–360_ checked by one-way ANOVA with Tukey’s post-hoc test. Significant difference of t_max_ checked by Friedmann’s test with Dunn’s post-hoc test: * (*p* < 0.05); ** (*p* < 0.01); *** (*p* < 0.001).

**Figure 9:**
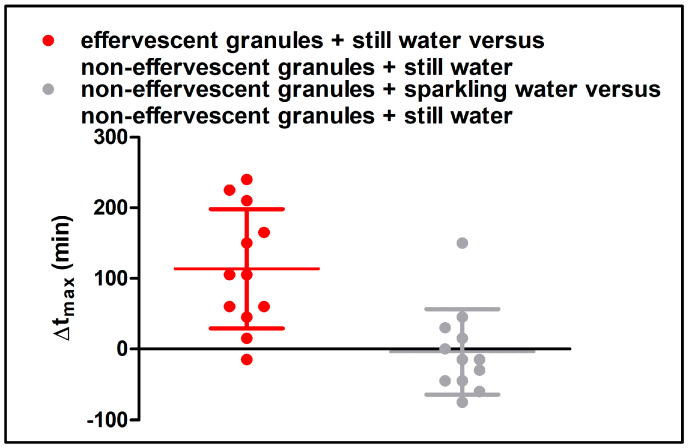
Difference of t_max_ (Δt_max_) between the administration of the effervescent granules with still water versus the administration of the non-effervescent granules with still water, and the administration of the non-effervescent granules with sparkling water versus the administration of the non-effervescent granules with still water (n = 12, mean ± standard deviation, points represent individual values).

In Figure 8, the statistical analysis of c_max_, t_max_, the AUC_0–120_, and the AUC_0–360_ is shown. A significantly higher t_max_ following the administration of the effervescent granules with still water compared to the application of the non-effervescent granules with still and sparkling water could be detected. As shown in Figure 9, in 11 of 12 subjects, the highest t_max_ was observed after the application of the effervescent granules with 240 mL still water. In contrast, the individual t_max_ values after the application the non-effervescent granules with still or sparkling were mostly very close to each other, and the higher values were distributed over both study arms. Unexpectedly, c_max_ and the AUC_0–360_ were also significantly greater following the administration of the effervescent granules with still water compared to the other two study arms. In contrast, the AUC_0–120_ tended to be smaller after the application of the effervescent granules with still water compared to the other two study arms. However, a statistically significant difference could only be detected in comparison to the administration of the non-effervescent granules with still water.

## 4. Discussion

In the present study, the effect of carbon dioxide on the absorption of the model drug caffeine was investigated. For this, an effervescent and a non-effervescent granule formulation were manufactured. The use of hot melt extrusion proved to be a reliable anhydrous manufacturing method for the granules. Hot melt extrusion has already been used to manufacture granules by other work groups [21]. Zaher et al. also produced effervescent granules containing pantoprazole and amitriptyline using PEG 4000 [22]. As PEG 4000 has a relatively low melting point (54 °C), the granulation process could be carried out at low temperatures. Therefore, thermic degradation of the ingredients was not of concern. The non-effervescent granules were produced in the same way and with the same ingredients, except for the absence of citric acid and sodium hydrogen carbonate. The granules showed a reproducible content of the model drug caffeine, as well as a fast disintegration and release of the drug in compendial dissolution tests. A water-soluble bag made from PVA as granule carrier was introduced to avoid oral contamination with caffeine and, in turn, in order to enable the measurement of salivary kinetics. This bag did not substantially delay the dissolution of the granules. The dissolution of the effervescent and the non-effervescent granules were very similar in the compendial tests, except for the dissolution of the granule carriers in phosphate buffer pH 6.8, where the effervescent granules showed a faster and higher drug release. The biorelevant dissolution test with the GastroDuo showed a slightly faster release of the effervescent granules compared to the non-effervescent granules. Most likely, this was attributed to the accelerated disintegration caused by the formation of carbon dioxide. Nonetheless, a fast disintegration in the stomach could be assumed for both manufactured granule formulations, as the thin polymer film bags opened rapidly in all performed dissolution tests.

Despite the fast release of both granule formulations in the compendial dissolution test, the rise of salivary caffeine concentrations differed significantly between the effervescent and the non-effervescent granules. For the non-effervescent granules, c_max_ was reached comparatively early, 123 ± 60 min after application with 240 mL still water and 119 ± 58 min after the application with 240 mL sparkling water, respectively. After the application of the effervescent granules with 240 mL still water, t_max_ was significantly delayed with 236 ± 55 min. 

**Figure 10:**
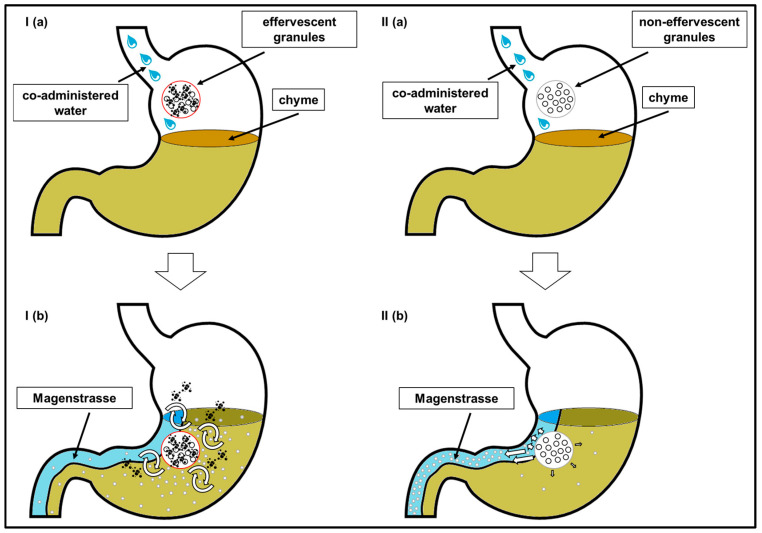
Scheme of the distribution of the model drug caffeine in the postprandial stomach following the administration of the effervescent granules bags with 240 mL still water (**I**) and the non-effervescent granules with still and sparkling water (**II**) before (**a**) and after (**b**) disintegration of the granule bags. The release of CO_2_ from the effervescent granules led to a pronounced mixing of caffeine with the chyme, followed by a slow calorie-dependent gastric emptying. In contrast, following the administration of the non-effervescent granules with still and sparkling water, more caffeine was distributed in the co-ingested water and emptied rapidly by the mechanism of the stomach road (*Magenstrasse*).

In Figure 10, the distribution of the model drug caffeine in the postprandial stomach following the ingestion of the granule bags is illustrated. In all study arms, a certain amount of caffeine was emptied from the stomach together with the co-administered water by the mechanism of the stomach road, apparent through a fast initial rise of salivary caffeine concentrations. However, obviously not the whole caffeine content left the stomach by this mechanism. As caffeine is highly water soluble and shows high permeability (BCS class I), its absorption rate is mainly determined by the gastric emptying rate. Thus, it can be used as marker substance for gastric emptying [12]. The emptying of water from the stomach follows first-order kinetics and is in the fed state as fast as in the fasted state. The time that is necessary for 240 mL to be emptied from the stomach was reported to be 15–45 min [8,9,10,11]. In all three study arms of the present study, c_max_ was reached markedly later. Apparently, some caffeine was mixed into the chyme regardless of effervescent behavior of the granules and carbonic acid content of the co-administered water. In contrast to water, food is emptied from the stomach at a much slower rate. The rate of gastric emptying of food is mainly dependent on its caloric content and reported to be 2–4 kcal/min. Koziolek et al. could show that the intragastric volume after the intake of the same high fat standard breakfast, as in the presented study, does not reach fasted state values even well beyond 6 h after the beginning of meal ingestion [8]. Thus, drug content that is mixed with the chyme is absorbed at a comparatively slow rate. If the model drug would have been distributed homogenously within the chyme, caffeine should have been emptied from the stomach and thus absorbed from the intestine for several hours. Obviously, the portion of caffeine that was distributed into the chyme was smaller after the application of non-effervescent granules with still and sparkling water than after application of the effervescent granules with still water. However, due to its hydrophilicity, caffeine can be washed out of the chyme by the gastric secretions that are released during digestion. 

**Figure 11:**
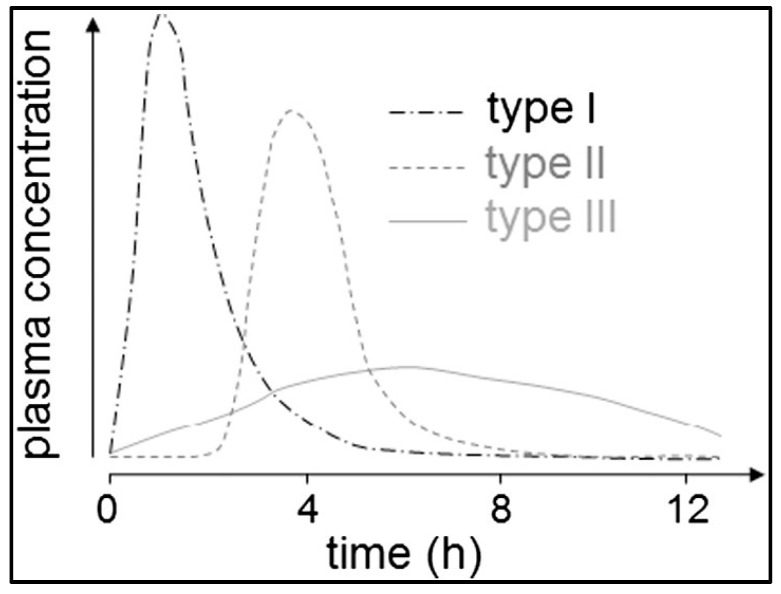
Proposed types of onset of plasma concentration after the administration of immediate release formulations in the fed state. Type I: rapid onset of drug plasma concentrations; Type II: rapid onset of drug plasma concentrations after a lag time; Type III: slow onset of drug plasma concentrations over several hours (reprinted from Koziolek et al. [7] with permission from Elsevier).

Koziolek et al. proposed three types of onsets of plasma concentration following the application of immediate release dosage forms in pharmacokinetic studies in the fed state as can be seen in Figure 11 [7]. Briefly, Type I plasma profiles can be characterized by a rapid onset of plasma concentrations caused by rapid disintegration of dosage forms close to the stomach road. For Type II profiles, a delayed onset of plasma concentration is typical, which is triggered by a phase of low mechanical stress due to the localization of the dosage form in the fundus, followed by a gastric emptying by the stomach road that is caused by the next water intake. The slow onset of plasma concentrations of Type III profiles can be explained by mixing of the drug content with the chyme, followed by a slow calorie-dependent gastric emptying.

As described earlier, several studies described an increase in gastric motility following the ingestion of sparkling water [2,3,4,5]. Additionally, Pouderoux et al. observed that the carbonation of water that was administered after a solid–liquid meal did not change overall emptying of gastric contents, but resulted in a more pronounced distribution of solid and liquid meal contents to proximal part of the stomach for the first hour after water ingestion [6]. The authors attributed this effect to a distention of the proximal stomach due to released gas volume of the sparkling water. 

The hypothesis of our work was that the induction of gastric motility through the release of carbon dioxide could promote the mixing of drugs into the chyme in the fed state, thus leading to a Type III onset of salivary caffeine concentrations by increasing the gastric residence time of the model drug caffeine. The manufacturer of the sparkling water used in this study (Gerolsteiner Brunnen GmbH & Co. KG) states that that it contains 1816 mg hydrogen carbonate and 7000 mg carbonic acid per litre [23]. Assuming complete conversion to carbon dioxide, a mass of 1506 mg carbon dioxide (equivalent to 837 mL at 25 °C and 101,325 hPa) would be released after administration of 240 mL. However, it has to be considered that a certain amount of the carbonic acid of the sparkling water being lost by opening the bottle could not be prevented. In comparison, the mass of 1500 mg granules per dosage form equals to 391 mg (26.07%) sodium hydrogen carbonate that would be converted to 205 mg carbon dioxide. In combination with the 100 mg carbon dioxide from the 240 mL still water (manufacturer specification 577 mg hydrogen carbonate per litre [24]), this would add up to 305 mg carbon dioxide (equivalent to 169 mL at 25 °C and 101,325 hPa). Thus, the motility inducing effect of carbon dioxide after the application of the non-effervescent granules together with sparkling water should have been at least as distinct as after the administration of the effervescent granules with still water. However, our results suggest that the postulated influence of the carbon-dioxide-induced increase in gastric motility is not the crucial factor for the prolonged gastric residence time of caffeine in the fed state. While the application of the non-effervescent granules with sparkling water did not lead to a significant change in t_max_, compared to the application with still water, the administration of the effervescent granules with still water showed the anticipated effect of a prolonged release from caffeine from the stomach. Obviously, the carbon-dioxide-releasing agent has to be incorporated into the granule formulation in order to enhance the gastric residence time of the drug. It is not clear whether this effect can be explained by increased gastric motility or if the released carbon dioxide induced the mixing by other means.

However, the results of this study cannot be interpolated to any (hydrogen) carbonate-containing immediate release dosage form. The release of carbon dioxide is a commonly used technique for a faster disintegration and dissolution of monolithic dosage forms. For example, the administration of a fast disintegrating and dissolving Aspirin^®^ tablet was shown to lead to more reproducible fast onset of acetylsalicylic acid with a higher c_max_ than the application of the regular Aspirin^®^ tablet, even under fed conditions [25]. The authors concluded that the fast disintegration led to a rapid gastric emptying of the drug together with the co-administered water by the stomach road. Similarly, a paracetamol formulation that contained 630 mg sodium hydrogen carbonate per tablet was also shown to produce a faster onset of plasma concentration under fasted and fed conditions, compared to a normal tablet [26,27]. For monolithic dosage forms, the disintegrating and dissolving promoting impact of carbon dioxide seems to be paramount for gastric emptying of the drug content, rather than the mixing due to the incorporated (hydrogen) carbonate. In contrast, the disintegrating and dissolving promoting impact of carbon dioxide is not as decisive for granular dosage forms, as the contained drug content can be dispersed prior to the formation of carbon dioxide. Subsequently, the formation of carbon dioxide seems to promote a local mixing of the already dispersed drug.

It has to be emphasized that the enhanced gastric residence time by the utilization of the effervescent granules only shows the targeted increase of the gastric residence time under fed conditions. In the fasted state, a rapid gastric emptying of the effervescent and the non-effervescent granules, together with the incorporated drug, could be assumed, as the granules would follow the co-ingested water. It is questionable whether the release of carbon dioxide following the administration of the effervescent granules could even lead to a faster onset of salivary caffeine concentration compared to the administration of the non-effervescent granules. Caffeine shows high water-solubility and both granules showed a rapid dissolution in the compendial dissolution experiments. Possibly, the release of carbon dioxide from the effervescent granules could lead to a more pronounced mixing of caffeine into the co-administered water; thus, to a faster onset of salivary caffeine concentrations. However, under fasted conditions, the carbonation of water does not have an effect on the overall gastric emptying of liquids [28]. Similarly, the carbonic acid content was shown to have no influence on the gastric emptying of glucose solutions following a fasting period of 10 h [29]. Thus, overall, it is likely that the effervescent and the non-effervescent granules show similar pharmacokinetics after administration in the fasted state.

The significantly higher c_max_ and AUC_0–360_ following the application of the effervescent granules were not expectable. The mean c_max_ following the application of the effervescent granules was about 1.2 times higher than in the other two study arms; the corresponding AUC_0–360_ was approximately 1.1 times higher. It is very unlikely that the effervescent granule bag increased the bioavailability of caffeine, as caffeine is almost completely absorbed from the gastrointestinal tract after oral ingestion. The observed difference of AUC_0–360_ of the different formulations is most likely related to more residues of caffeine from the non-effervescent granules that remained in stomach. During the sampling time, this fraction of applied dose was not absorbed, thus decreasing AUC_0–360_. In our opinion, an effective discussion of the bioavailability based on the AUC_0–360_ is not feasible. For this, the AUC_0–ꚙ_ should have been determined, which was not possible based on the rather limited observation period (6 h) in our study. 

Overall, the administration of sparkling water did not result in an altered gastric emptying of caffeine dosed under fed conditions as a multiparticulate immediate release formulation. However, under fasted conditions, van den Abeele et al. found a faster onset of plasma concentration following the administration of a paracetamol tablet together with carbonized water [2]. It has to be emphasized that the results of our study and those of the study of van den Abeele et al. are not directly comparable. Firstly, the administrations were carried out in different prandial states. As described earlier, several physiological parameters, like gastric emptying, differ between the fasted and the fed state. Secondly, van den Abeele et al. used a tablet, which represents a monolithic dosage form, while our granules are a multiparticulate formulation. Thirdly, the types of the co-administered water, and thus probably the carbonation grade, were different. While the sparkling water used in the present study (Gerolsteiner Sprudel) contains 7 g carbonic acid per litre, van den Abeele et al. made no statement on the content of carbonic acid in the sparkling water they used (Chaudfontaine^®^). Thus, the accelerating effect of carbonized water on paracetamol absorption in this study is probably mainly attributed to an acceleration of disintegration and dispersion of the tablet, and most likely not related to a faster gastric emptying rate.

The administration of the effervescent granules showed the anticipated prolongation of gastric residence of the incorporated model drug caffeine. Thus, the application of effervescent granules under fed conditions represents a promising approach to increase the gastric residence time of drugs. This could be beneficial for several therapeutic approaches, where a continuous absorption of the drug in the small intestine aimed for or where a local treatment of the stomach shall be achieved.

## 5. Conclusions

In the present study, the effect of carbon dioxide on the gastric emptying of the model drug caffeine under fed conditions was investigated. Our hypothesis was that the release of carbon dioxide would promote the mixing of caffeine into the chyme, which would result in prolonged gastric residence and a slower onset of salivary caffeine concentrations. The administration of the non-effervescent granules with sparkling water did not result in significant differences in salivary caffeine pharmacokinetics in comparison to the administration with still water. In contrast, the onset of salivary caffeine concentration was significantly slower following the administration of the effervescent granules with still water. It seems that the carbon dioxide had to be incorporated into the granule formulation in order to promote a mixing of drugs into the chyme. Sparkling water did not show the same effect towards a prolonged gastric residence of caffeine. Thus, it is questionable whether the observed mixing was caused by carbon-dioxide-induced motility. Or rather, an intensive local dispersion of an already dispersed formulation into the chyme due to local effervescent and a fluid dynamic effect related to spontaneously and rapidly formed gas bubbles.

## Figures and Tables

**Table 1:** Composition of the effervescent and the non-effervescent granules.

Ingredients	Quantity (%)	
	Effervescent Granules	Non-Effervescent Granules
Caffeine	6.67	6.67
Sodium hydrogen carbonate	26.07	0.00
Citric acid, anhydrous	9.93	0.00
Microcrystalline cellulose (MCC)	20.00	43.99
Polyvinylpyrolidone (PVP)	2.00	2.00
Mannitol	8.67	8.67
Macrogol 4000	20.00	20.00
Lactose monohydrate	6.67	18.67

**Table 2:** Demographic data of the volunteers (n = 12).

Parameter	Median (Range)	Mean ± SD
Sex	m:8; f:4	-
Age	26 (23–30)	26.0 ± 2.0
Height (cm)	183.5 (165–205)	182.7 ± 12.3
Weight (kg)	79 (64–99)	79.6 ± 12.5
BMI (kg/m^2^)	23.2 (21.4–26.9)	23.8 ± 2.0

## Data Availability

Data are contained within the article.
